# Sex matters: The effects of biological sex on adipose tissue biology and energy metabolism

**DOI:** 10.1016/j.redox.2017.04.012

**Published:** 2017-04-13

**Authors:** Teresa G. Valencak, Anne Osterrieder, Tim J. Schulz

**Affiliations:** a Institute of Physiology, Pathophysiology and Biophysics, Department of Biomedical Sciences, University of Veterinary Medicine Vienna, Veterinärplatz 1, A-1210 Vienna, Austria; b Department of Biological and Medical Sciences, Faculty of Health and Life Sciences, Oxford Brookes University, Gipsy Lane, Headington, Oxford OX3 0BP, UK; c Department of Adipocyte Development and Nutrition, German Institute of Human Nutrition Potsdam-Rehbrücke, 114-116, Arthur-Scheunert-Allee, 14558 Nuthetal, Germany; d German Center for Diabetes Research (DZD), 85764 München-Neuherberg, Germany

**Keywords:** Body fatness, Adipose tissue, Sex-specific differences, Adipokines, Adipocytes, Obesity, Energy metabolism

## Abstract

Adipose tissue is a complex and multi-faceted organ. It responds dynamically to internal and external stimuli, depending on the developmental stage and activity of the organism. The most common functional subunits of adipose tissue, white and brown adipocytes, regulate and respond to endocrine processes, which then determine metabolic rate as well as adipose tissue functions. While the molecular aspects of white and brown adipose biology have become clearer in the recent past, much less is known about sex-specific differences in regulation and deposition of adipose tissue, and the specific role of the so-called pink adipocytes during lactation in females. This review summarises the current understanding of adipose tissue dynamics with a focus on sex-specific differences in adipose tissue energy metabolism and endocrine functions, focussing on mammalian model organisms as well as human-derived data. In females, pink adipocytes trans-differentiate during pregnancy from subcutaneous white adipocytes and are responsible for milk-secretion in mammary glands. Overlooking biological sex variation may ultimately hamper clinical treatments of many aspects of metabolic disorders.

## Highlights

• Sex differences of endocrine and metabolic function in adipose tissue.
• Sex-specific differences in regulation and deposition of fat are often overlooked.
• Pink adipocytes trans-differentiate from white fat cells in pregnant females.
• Gender-specific medicine: implication for therapeutic approaches in metabolic disease.

## Graphical abstract

**Figure:**
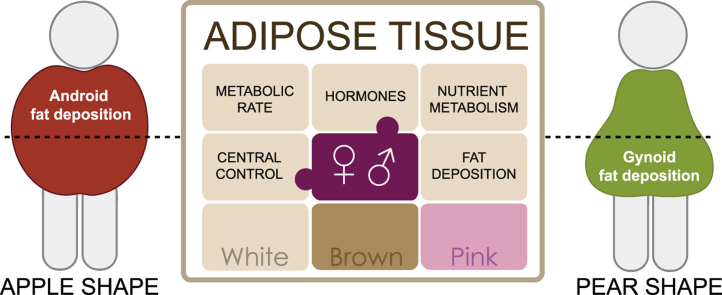

## 1 Introduction

Adipose tissue is a complex, essential and highly active metabolic and endocrine organ widely recognized to fulfil a variety of functions. These include mechanical protection and thermic insulation, regulated storage and release of energy, immune responses, and non-shivering thermogenesis [1], [2], [3]. In the recent decades, excess adipose tissue accumulation (obesity) has emerged as one of the major medical challenges in societies worldwide. Obesity leads to overall increased incidences of metabolic complications, such as insulin resistance and diabetes, dyslipidaemia and cardiovascular dysfunction, but also neurodegenerative diseases and cancer [4], [5], [6], [7]. Adipose tissues, as component of connective tissues, occur as depots either under the skin (subcutaneous depots) or in the visceral cavity, where they surround the inner digestive organs and thus are considered a multi-depot organ [2]. Excess white adipose tissue in overweight or obese patients is generally seen as risk factor, and this risk association is strongest in obese patients with visceral adiposity [8].

**Fig. 1:**
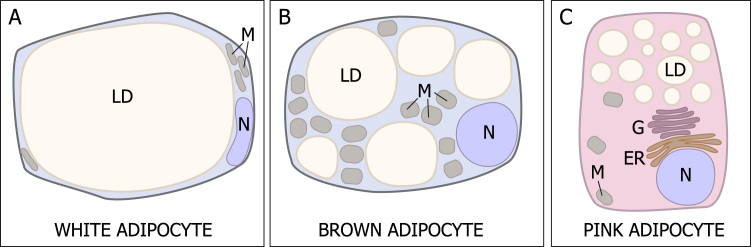
Schematic general cytology of the three cell types constituting the adipose tissue organ: white, brown and pink adipocytes. LD=Lipid droplet, M Mitochondrion, N Nucleus, G Golgi Apparatus, ER Endoplasmic reticulum. (For interpretation of the references to color in this figure legend, the reader is referred to the web version of this article).

The general cytology of the key functional units of adipose tissue, the adipocytes, is well understood [2], [3], [9] (Fig. 1). White and brown adipocytes differ clearly in their physiology and structural characteristics [2], [3], [9]. While brown adipocytes transform energy for thermogenesis, e.g. the production of heat, white adipocytes store and release energy according to the metabolic needs of the organism [1]. In the recent past, an intermediate type of brown-like fat cells, the so-called beige [10] or brite (brown-in-white) [11] adipocytes, has been described. These cells occur interspersed within depots of white adipose tissue (WAT), and trans-differentiate from mature white adipocytes [12], [13], or may also, to some extent, derive from de novo differentiation of progenitors [14]. Beige/brite adipocytes develop mostly in response to cold exposure or other mechanisms of adrenergic stimulation of the WAT depots [15], [16]. A female-specific cell type, the so-called pink adipocyte, arises during pregnancy from subcutaneous depots. It produces and secretes milk and plays a key, if transient, role in the female metabolism during the early stages of offspring fostering [17] (Fig. 1). Just as white adipocytes trans-differentiate into beige adipocytes during browning, white adipocytes also transform into milk-producing pink adipocytes in pregnant females [13]. As indicated in Fig. 1, pink adipocytes are milk-secreting alveolar glands that are quickly re-constituted into regular white adipocytes after weaning. Not surprisingly therefore, once the mammary glands have re-shaped into subcutaneous adipocytes again, the energy demands of the female returns to baseline. In humans, postpartum weight retention however is very common, and often gives rise to long-term overweight and obesity [18].

**Fig. 2:**
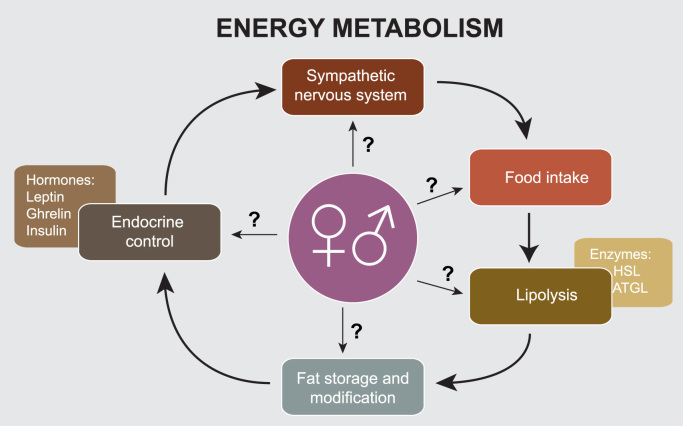
Feedback loop scheme involved in the regulation of body fatness. Sex-specific differences may impact on each of the given factors.

A substantial body of knowledge has been assembled in relation to maladaptive pathologies, adipose tissue biology and obesity, and has been reviewed in great detail before [19], [20], [21], [22]. However, one area that has received comparatively little attention is the transferability of research results between different sexes of the same model organism and sex-specific metabolic functions of lipids metabolism, e.g. the process of lactation. Differentiation between sex-specific metabolic differences that translate into altered distribution of fat stores as well as mobilization of lipids in response to metabolic challenges may help to better understand the contemporary obesity epidemic. This review aims to summarize the current knowledge on sex-specific differences in adipose tissue energy metabolism, focussing on mammalian model organisms as well as human-derived data. We also discuss the sex differences in nutrient homeostasis as well as metabolic hormone/adipokine regulation (Fig. 2).

## 2 Adipose tissue energy metabolism

Mammalians have evolved a precisely tuned endogenous system controlling energy balance, i.e. energy/food intake and expenditure. This involves local processes in the peripheral adipose organs, such as lipid mobilization and storage, as well as release of endocrine signals in the form of hormones and other signalling molecules. On the other hand, central mechanisms integrate food intake regulation, energy expenditure and environmental cues with such endocrine signals to achieve metabolic balance (Fig. 2). Several models have been put forward to explain this elaborate control system (summarised in [23]). The set point model proposes the existence of an active feedback loop that determines overall fat mass of the body, thus linking energy stores in adipose tissue to expended energy using a pre-determined level that presumably is set in the brain [23]. On the molecular level, this model system proposes the existence of a fat-derived signal, a lipostat, that is sensed by the brain to control influx of nutrients, e.g. hunger, or outflow of excess nutrients for instance by thermogenic dissipation [24]. In the more recent past, the hormone leptin, secreted from adipocytes, has been introduced as a potential mediator of the set point [25], [26], [27]. While this model is suitable to explain most biological aspects of body fatness, it is difficult to incorporate extrinsic aspects, such as societal and sociological cues, that also contribute to the development of adiposity [20].

As an alternative, the settling point hypothesis suggests a passive feedback between the size of the fat depots and energy dissipation [23]. Here, the level of energy influx also determines the outflow of energy, and has been likened to the more or less stable water levels of a lake, where the extent of water leaving is equal to the water addition from the environment. Both of these models, however, fail to explain the gene-by-environment interactions, which are the key to a constant body mass [23]. Thus, two additional models, the general intake model and the dual intervention point model are currently tested [23]. These two models provide an evolutionary rationale to explain why harmonised systems to control energy intake and expenditure might have evolved. The dual intervention point model [28] in particular proposes a lower and an upper intervention point for body fatness predicted by the risk of starvation on the one hand, and by the risk of falling prey to a predator due to immobility on the other hand [28]. To date, the aspect of biological sex has not yet been addressed in context with the above-enumerated models on the regulation of body fatness. We envisage that the models may be applicable to both sexes similarly. However, the exact localization of the upper and lower intervention point may be somewhat shifted in females due to the outstanding energy demands during lactation (see subchapter on lactation).

Key to the fine tuning of energy and food intake and providing the basis of all the above-mentioned models is the interplay of the three metabolic hormones leptin, ghrelin and insulin [29], and a number of other important regulatory signals. Leptin and its receptor are innately connected to adipose tissue function due to their ability to convey information on the nutritional status and to modulate metabolic rates accordingly [29]. Leptin was among the original cytokines to be identified as adipokines, e.g. endocrine signals secreted from adipose tissue [30], and is now recognized as a major regulator of energy homeostasis [29], [31], [32]. Since then, a wide range of new adipokines with diverse biological effects have been identified, reshaping the traditional role of adipose tissue depots as energy storage units to major endocrine organs [32], [33]. Similarly, the gastrointestinal peptide, ghrelin, which was discovered in 1999, plays a key role in feeding behaviour and metabolism [34], [35]. Finally, pancreatic islet-derived insulin drives rates of lipolysis and triacylglycerol uptake from the blood into adipose tissue and skeletal muscles and therefore is the third major player in the regulation of body fatness (Fig. 2).

## 3 Sex-specific differences in adipose tissue distribution patterns and metabolic control

Traditionally, the net amount of body fat is considered in diagnosing body fatness in mammals, which in humans is reflected by the computation and importance of the body mass index (BMI), [36]. The distribution of adipose tissue depots has recently received a more differentiated level of attention. While visceral (and abdominal-subcutaneous) fat is associated with features of the Metabolic Syndrome, such as type 2 diabetes, cardiovascular disease, atherosclerosis and hypertension, fat accumulation in subcutaneous gluteal-femoral depots of body fat may be protective against mechanical loads as well as buffering during times of food scarcity [37]. As such, the distribution of adipocytes in specific depots is far more important for the pathogenesis of adiposity and associated diseases than purely quantitative considerations of body fatness [37], [38].

Aside from the well-known pathophysiological consequences of an unhealthy distribution or accumulation of adipose depots, the perceived ideal of a beautiful body in many cultures favours little or no accumulation of visible fat mass. Surprisingly, in a recent epidemiological study of ten populations from diverse African, Asian and Caucasian backgrounds, an inverse correlation between body fatness or BMI and subjective perception of physical attractiveness was uncovered [39]. As adipose tissue is mostly perceived as unattractive and harmful, and even indicative of an intemperate and unhealthy lifestyle, it has received little scientific attention until some decades ago, when it was identified as a vital organ in vertebrate physiology [31], [32], [40]. Based on the assumption that greater body fatness may reflect a higher potential to survive famines and/or produce viable offspring, it was suggested that increased body fatness may therefore signal evolutionary fitness, a prediction that does not hold true [39]. Aesthetics aside, recent studies suggest an optimum adipose tissue quantity, which is thought to assist in surviving periods of energy shortage, illness or other situations during which fat depots buffer the energy needs of the organism [40], [41]. Accordingly, absence or complete lack of adipose tissue reduces fertility and survival rates in almost all stressful and energetically challenging conditions (summarised in Table 1). Interestingly, recent analyses have found that a certain level of mild overweight may even be beneficial in relation to all-cause mortality and life expectancy [42].

**Table 1:** Main functions of adipose tissue in mammals.

*Physiological state*	*Tissue function*	*Location in body*	*References*
Reproduction, Lactation, Survival of energy shortages, Age/Frailty, Disease	Energy storage	All white depots, Visceral,	[1], [114]
		Subcutaneous	
Non-shivering thermogenesis	Source of heat	Supraclavicular,	[114]
		Paravertebral,	
		Cervical,	
		Interscapular,	
		Axillary,	
		Mediastinal	
Temperate and polar regions: Marine animals	Thermal insulation	Subcutaneous	[1]
		(“blubber”)	
Metabolism, Starvation, Food intake, Digestion, Endocrine control	Production of hormones, Signalling molecules	All depots,	[41], [62]
		Visceral,	
		Subcutaneous	
Mechanical load	Support, Buffering capacity	Subcutaneous,	[115]
		Epicardial	

Females and males differ quite markedly in adipose tissue distribution, but the biological underpinnings of these associations remain to be investigated in more detail [38]. While the android fat deposition refers to fat accumulation in the upper body and upper abdominal areas, i.e. above the waist, also known as the so-called apple shape in obese men, the gynoid fat deposition is described as accumulation of body fat below waist around the hips and thighs, i.e. the so-called pear shape [38]. It has been proposed that circulating gonadal steroids determine these sex-specific differences in adipose tissue distribution, which can be observed even after menopause, but are much more pronounced during the reproductive phase [43]. However, sex specifics already occur before puberty, suggesting that at least some mechanisms of differential adipose tissue distribution are unrelated to sex steroids [44], [45]. Interestingly, and apart from gonadal steroids, the number of X chromosomes also contributes to sex-specific differences in obese mice [46], and in 47, XYY men a trend to elevated central adiposity is observed [47]. It should be noted that there is no equivalent android (apple) or gynoid (pear) shape body fat distribution types in laboratory rodents, even though differences in adiposity between the sexes are well documented [43].

To date, evidence is accumulating that sex-specific differences in the functionality of each type of adipose tissue are not solely based on the size of a specific depot (e.g. visceral vs. subcutaneous fat), but also on differential gene expression patterns between the sexes leading to different proteins expressed in, and potentially secreted form, visceral and subcutaneous adipose tissues [38], [43]. The epidemiological and clinical evidence has clearly established a differential risk association with high risk for metabolic disease in the android/apple shaped obesity-type, whereas gynoid/pear-shaped adiposity appears to show less association or may even exert protective effects [48], [49]. For instance, correction for visceral adipose accumulation in men and women essentially abolished sex-specific differences in the development of cardiovascular disease [50], and preferential accumulation of adipose tissue in peripheral and gluteofemoral depots protects against development of atherosclerosis [51], [52], [53]. In summary, a fat distribution pattern more pronounced in female individuals contributes to a healthier metabolic profile and may even be involved in the determination of overall life expectancy, which is well known to be elevated in women compared to men. The physiological consequences of excess or insufficient fat storage remain incompletely understood in many regards. One of the key remaining questions is to determine the limitations to metabolic flexibility that either allow appropriate handling of dietary nutrients or lead to failure to do so, which will ultimately lead to obesity and metabolic disease. Most important in this context is the apparent and prominent difference in adipose tissue distribution between men and women, colloquially referred to as the apple- and the pear-shaped obesity [38]. To highlight sex-specific differences pertaining to the most important functions of adipose tissue, we compiled the available data from mammals depending on physiological states and by referring to different adipose depots and types in the body (Table 1). Adipose tissue thus is key during phases of net energy expenditure such as reproduction and famines, during disease and frailty, during cold when body temperature needs to be conserved and also as an outer barrier against mechanical stress (Table 1).

In the last two decades, white adipose tissue also emerged as “a pivotal organ controlling lifespan” [54]. Numerous target genes relating to adipose tissue metabolism to date are supposed to be involved in aging [54], [55]. The link between aging and adipose tissue is intuitive as body mass increases with age, particularly in the post-reproductive age [56], [57]. In old age, body fat and accordingly the entire phenotype of gene expression and metabolomics is changed [58]. Among possibly hundreds of other genes, sirtuin 1 (SIRT1), ubiquitously expressed and present in white adipose tissue, was shown to down-regulate the stress-responsive inducers of cell death (p53), apoptosis and the forkhead family of transcription factors (FOXO) during caloric restriction [54]. Similarly, tissue-specific knockout of the insulin receptor also induces lipolysis and extends lifespan in mice [59]. Thus, in order to delay aging, a reduction in white adipose tissue seems to be the general recommendation although body fat mass of senescent mammals shows complicated dynamics [55], [60]. For the life-shortening effects of excess white adipose tissue both the modification of white adipose tissue derived adipokines and the higher risk for age-related metabolic diseases such as type 2 diabetes were made responsible [54], [55]. However, recent exciting research also reveals that white adipose tissue, lipolysis and lipolytic enzymes play a key role for survivability of cancer [61]. Cachexia is a life-threatening condition of dramatic loss of adipose tissue as observed in late-stage cancer patients with a limited survival prognosis [61]. Absence of ATGL and HSL, however, delayed the development of cachexia [63], suggesting that reduced ability for lipid mobilization may be a suitable approach to attenuate progression of cachexia. The role of sex-specific differences in development of cachexia related to malfunction of the lipolytic enzymes still needs to be identified.

Obese female mice contained more adipose tissue mass compared to weight-matched males, but were more glucose tolerant, displayed elevated expression of adiponectin and reductions in adipose tissue immune cell infiltration as well as oxidative stress levels. This observation suggests that the protective benefits of oestrogen on oxidative stress is retained during adiposity and may help to ameliorate metabolic defects in female compared to male mice [64]. Along these lines, it was previously recognized that oestrogen itself has antioxidant properties and induces expression of several longevity genes, which may in turn help to promote stress resistance [65]. In summary, the well-known effect of longer life expectancy may at least in part rely on metabolic differences and may particularly pertain to molecular and functional sex differences in adipose tissue depots.

## 4 Sex-specific differences in endocrine function and adipokine secretion

In view of the above-mentioned major contributors to metabolic control, it is not surprising that the scientific progress in endocrinology raised the status of white adipose tissue from a passive repository of excess lipids to a key endocrine organ [31]. When discussing the wide array of secreted molecules acting as adipokines, it should not be overlooked that indeed fatty acids are quantitatively the most important secretory products of adipose tissue.

Fatty acids per se have been linked to lipotoxicity, and are therefore usually metabolised rapidly to minimize this potentially pathogenic effect [66]. In women, non-oxidative FFA disposal rates were significantly increased compared to men [67]. Noradrenaline derived from the sympathetic nervous system drives the breakdown of triacylglycerols into free fatty acids and glycerol during lipolysis, which is activated by cold exposure, caloric restriction or other situations characterized by increased metabolic demand for energy equivalents [68]. In light of the potential involvement of lipostat signals in setting metabolic rates and energy intake, one needs to consider nutrient sensing on all levels, e.g. in peripheral organs as well as in the central nervous system. Metabolic pathways involved in nutrient sensing play a critical role and are extensively reviewed in [69].

While substantial research is conducted to elucidate the molecular steps of the lipolysis pathways [62], and to identify new adipokines and their function, only limited attention has been paid to sex-specific differences of these adipose tissue derived factors. Researchers tend to avoid the use of female animals in the biomedical sciences for reasons around potentially higher metabolic variabilities related to the female hormonal cycle [70]. Analyses of sex-specific differences in circulating levels of some adipokines have revealed important differences. For instance, circulating levels of the hormone leptin are increased in women compared to men [71], [72], whereas male mice show higher leptin concentrations compared to female mice [73], [74], suggesting that some relationships between adipokines and energy metabolism as seen in females do not hold true in males and vice versa (Table 2). Another prominent example is adiponectin, a more recently discovered adipokine, primarily produced by subcutaneous white adipose tissue and inversely correlated to overall fat mass and metabolic perturbations [75]. As subcutaneous white adipose tissue is the main source for adiponectin, it is also thought to be the reason why there are no or little detrimental health associations for people with a high degree of subcutaneous adiposity [48], [49]. Ever since its discovery in 1995, this adipokine has repeatedly been associated with improved metabolic outcomes such as enhanced insulin sensitivity and reduced risk for cardiovascular complications in obese patients [76], [77]. Adiponectin levels are higher in females than in males for so far unknown biological reasons [75]. Similarly, it was found in adipose tissue biopsies that adipocyte size, basal lipolysis and fatty acid oxidation rates as well as exercise driven lipolysis differ significantly between men and women [78]. From studies in humans, differences in fatty acid mobilization, oxidation and also storage are well documented [37], [78]. Moreover, cyclical reproductive hormones do not correlate well with adipokine levels [79].

**Table 2:** Sex- specific secretion of adipokines in humans, summarised from [37].

**Adipokine**	**Abbreviation**	**Women**	**Men**	**Role of site-specifity and adipose tissue vs. circulating**
Leptin		↑	↓	
Adiponectin		↑	↓	
Retinol-binding protein 4	RBP-4	?	?	
Plasminogen activator inhibitor-1	PAI1	?	?	
Dipeptidyl peptidase-4	(DPP)-4	?	?	
Chemerin/ Retinoic acid receptor responder 2	RARRES2	↑	↓	Site-specific differences
Lipocalin 2/neutrophil gelatinase-associated lipocalin	24p3	↑↓ ←→	?	Site-specific differences
Glypican-4	Gpc4	↑	↓	Site-specific differences
Omentin		↓	↑	Site-specific differences
Secreted frizzled-related proteins	SFRPs	↑	↓	
Vaspin (visceral adipose tissue-derived serine protease inhibitor)	Serpin A12	↑	↓	Adipose tissue≠ circulating levels

In light of these facts, failure to consider sex-specific differences in adipose tissue and its pathophysiological complications are potentially significant. A rather marked male study subject bias is well described, with an average of 3.7 males to one female subject contributing to overall scientific knowledge [70]. A prevalent explanation among researchers is the higher variability in metabolic parameters being affected by cyclical fluctuation of reproductive hormones, potentially leading to a more complicated experimental set-up and more complex and difficult interpretation of data [70]. Other reasons may range from matters of convenience, to reluctance to accept sex-specific effects, and finally additional costs arising from larger sample sizes. However, the assumption that female mammals are innately more variable than males seems not fully appropriate [80]. Notably, inclusion of women in clinical trials was mandated only in 1993 [80], and research on female animals or female-derived cell systems was not expressly sanctioned by the NIH until 2014 [81]. As impressively summarised by Beery et al. [80], sex-specific differences in pharmacokinetics and pharmacodynamics of many clinical therapies already affect women's health, showing the importance of following the recommendation to pursue a gender-balanced research strategy [80]. In summary, the differences in adipose tissue distribution between women and men, as discussed in the previous chapter, and the different survival probabilities of obese women and men [82], [83], make consideration of both sexes an important necessity and cannot be overlooked to the same extent as in rodent studies where sex differences may be more subtle and less apparent. Not surprisingly therefore, comprehensive reviews on human white adipose tissue metabolism exist [37], [77] that discuss information on the sex-specific secretion of the most important adipokines.

## 5 Sex-specific differences in brown adipose tissue and regulation by steroid hormones

Apart from its role in energy storage (Table 1) [1], adipose tissue serves as a source of heat to maintain body temperature and for thermal insulation. Its low specific weight and the high-energy yield of triglycerides compared to carbohydrates and proteins render it the favoured storage form for energy equivalents in the evolution of modern species. Not surprisingly therefore, small mammals below 20 kg body mass rely on physiological measures such as lower metabolic rates by means of torpidity to utilize less body fat, and still survive winter or periods of food scarcity.

Brown adipocytes with more fragmented, e.g. multilocular, lipid droplets and numerous mitochondria make up the thermogenic component of this type of adipose tissue. Mediated by uncoupled protein 1 (UCP-1), a protein uniquely expressed in brown as well as beige/brite adipocytes, substrate oxidation is uncoupled from ATP synthesis, and heat is released instead [2], [3], [16], [84], [85]. In a process generally considered a white-to-brown trans-differentiation of adipocytes, commonly referred to as browning, the recruitment of beige cells under in vivo conditions takes place in response to cold or adrenergic stimulation [16], [84]. In terms of sex differences, female mice are more responsive than males to browning of abdominal and subcutaneous white adipose tissue during β-adrenergic stimulation [86]. In humans, a greater abundance of BAT in women was reported early on [87], [88]. Consistently, female rats display higher levels of thermogenesis in ad libitum feeding conditions [89]. Of note, sex specific differences in BAT were reported for its lipid composition, while WAT depots showed a much less pronounced dimorphism [90]. Under caloric restriction, female rats deactivate facultative thermogenesis to a greater degree than males, which results in increased conservation of metabolic organ mass, and which has been proposed to ultimately be advantageous for female survival in food-limited situations [91].

While metabolic rates appear to be similar in women and men when adjusted to lean mass [38], the level of oestrogen-dependent sympathetic innervation was higher in females than in males [86], altogether suggesting that females are able to adjust their ability for thermogenesis more acutely than males. This sexual dimorphism seems to be due to differences in several aspect of brown adipocyte function that occur in female compared to male brown adipocytes. For instance, mitochondrial function, as indicated by increased cristae height and density in mitochondria of female BAT, and sensitivity of brown adipocytes to beta-adrenergic stimulation, was higher in females compared to males [92]. This is further corroborated by the observation that expression of thermogenic genes is lower in BAT of males fed a high fat diet than in females [91]. On the molecular level, expression of the major sex steroid receptors, e.g. androgen, oestrogen and progesterone receptors, have been documented on brown adipocytes [94]. Moreover, the actions of sex steroids on the nervous system have also been proposed as mediators of sexual dimorphisms in energy balance [95]. Oestrogen receptor (ER) knockout mice display lower metabolic rates and are obese, while ovariectomised mice become obese and display BAT atrophy [96], [97], [98]. These phenotypes could potentially be mediated by changed central signalling, and thus indirect effects of this hormone of BAT [95].

Similar to ER-deficient mice, androgen receptor (AR) knockout mice become obese, which is likely due to lowered energy expenditure and reduced expression of thermogenic genes such as UCP1, which incidentally contains an androgen-response element in its promoter [99]. In the case of androgen signalling, however, the available scientific evidence is complex: Some studies have suggested a negative regulatory role on UCP1 expression as in vitro treatment with testosterone decreased UCP1-expression [100], while no effect on thermogenesis was observed in animals treated with testosterone [101]. In summary, the role of AR in brown adipogenesis is not fully understood, and the literature suggests that direct aspects on brown adipocytes as well as control of food intake-dependent effects on metabolism [95]. Lastly, progesterone stimulates expression of UCP1 and lipolysis rates, and enhanced mitochondrial biogenesis [100], [102], [103]. Taken together, steroid hormones appear to play an important role in brown adipocyte metabolism and may well mediate the sexual dimorphisms observed in this adipose tissue type.

## 6 Lactation as female-specific aspect of lipid/energy metabolism: specialised demands on female metabolic energy balance and the identification of pink adipocytes

White adipose tissue is the most efficient depot of triglyceride storage. During the process of lipolysis, three main enzymes regulate the mobilization of free fatty acids from triacylglycerols stored in cellular lipid droplets (Fig. 2, Table 2): hormone sensitive lipase (HSL), lipoprotein lipase (LPL) and adipose triglyceride lipase (ATGL), (reviewed in [62]). HSL is located directly on the surface of the lipid droplet, and is stimulated by catecholamines such as the hormone epinephrine, depending on the physiological state [62]. Sex-specific differences in the expression of HSL were reported previously in skeletal muscles, where women were found to have higher intramuscular triacylglycerol during exercise than men, and also higher mRNA levels of HSL in the muscle [104]. Yet, as HSL activity during prolonged exercise is higher in men than in women, it is likely that enzyme-substrate interactions differ between the sexes [104]. Sex-specific differences in sustained and peak metabolic rates in mammals make great sense in the light of their evolutionary ecology, as females are the ones supporting offspring with milk, the major and unique source of energy for new-born mammals, whether monotreme, marsupial, or eutherian [105]. Maternal milk secretory products of the mammary gland involve multiple casein proteins, milk lipids i.e. milk fat globules, and milk sugars, i.e. lactose [105]. Milk was shown to have an ancient evolutionary origin of ca. 310 million years ago and is unique to mammals, although its composition is highly variable in different species [105], with fat contents ranging from a few per cent in rhinoceroses to around 60% in ice-breeding seals [106].

Conceivably, by far the most energy-demanding phase known from mammals is lactation, when females reach sustained metabolic rates about 6–8 times higher than resting levels, while ingesting food at peak rates and synthesising milk to support the young [17], [107], [108]. To meet these high energy demands over protracted periods of several weeks or even months, many mammalian species store adipose tissue prior to conception or during gestation for use later during reproduction [108]. Apart from being energy demanding, lactation comes with another side effect, the generation of substantial amounts of surplus metabolic heat [109], which needs to be balanced by increased heat loss. Hyperthermia during lactation may elevate the female's body temperature by 0.5–1.5° above non-reproductive levels, indicating the significant impact of the milk production process on overall energy balance [109]. In view of all this it is not surprising that during lactation, brown adipose tissue atrophies, lowered mitochondrial content, and a reduced gene expression of Ucp-1 were reported [109], [110]. Similarly, the down-regulation of UCP-1 driven thermogenesis observed during lactation has mainly been attributed to a decrease in the sympathetic nervous system activity to mediate suppression of thermogenesis by brown adipose tissue [111]. Along these lines, it was suggested from a study conducted in rats that a lower progesterone response during lactation plays a role in the impairment of non-shivering thermogenesis during lactation [112]. Meanwhile it has become quite clear that sex steroid receptor expression profiles in brown adipose tissue differ markedly between males and females, and that understanding those discrepancies in brown adipose metabolism will require studying both sexes [94].

The down-regulation of non-shivering thermogenesis is not the only metabolic adaptation in anticipation of and during lactation: Trans-differentiation of subcutaneous white adipocytes into milk producing alveolar glands takes place already during mid-pregnancy [13]. Lineage tracing has revealed that white adipocytes convert into alveolar epithelial cells during gestation but that they back-transform rapidly into normal adipocytes after weaning of the offspring, indicating a very high level and a new dimension of adipose tissue plasticity [13]. As the colour of the adipose tissue organ during gestation is pink, Giordano and colleagues proposed the label of pink adipocytes, also in reference to the brown/beige/brite nomenclature of thermogenic fat cells [17]. More recently, the researchers observed that post-lactational pink adipocytes may trans-differentiate into brown adipocytes, indicating further and unexpected plasticity of adipose tissue [113]. Clearly, further research is required to identify the destiny of milk producing alveolar glands in the metabolism of post-reproductive females. In view of the well-known problem of body weight control and energy balance after childbirth and lactation [18], we are proposing to consider the recent discoveries from adipose tissue functional anatomy.

## 7 Summary

Here, we summarize the prevalence of sex-specific differences in human metabolic disease and adipose tissue biology, and the potential arguments leading to an apparent reluctance to conduct research on both sexes in human patients as well as laboratory animals. Ignoring such sex-specific biological variation may ultimately hamper progression in the treatment of many aspects of metabolic pathophysiology and may restrain the concept of gender medicine, which has received significant attention in the recent scientific literature. Other areas that may be related to sex-specific differences in adipose tissue function may pertain to the microbiome, which has become a recent focus in many areas of research. Similarly, the interactions of adipose tissue, sex and the aging process have not been investigated in great detail, and clearly warrant further investigation. Ultimately, improved understanding of female adipose tissue biology in the same level of detail as is currently the case in male organisms could not only lead to gender-specific equality in medical treatments, but also to a more broad understanding of the pathological understanding of metabolic diseases in general.
